# Effect of Oral Application of Povidone-Iodine on the Amount of Viable Bacteria Entering the Lower Respiratory Tract in Intubated Patients With Oral Cancer: A Preliminary Study

**DOI:** 10.7759/cureus.72240

**Published:** 2024-10-23

**Authors:** Akira Imakiire, Sakiko Soutome, Kei-ichiro Miura, Nanako Ito, Mirai Higaki, Fumitaka Obayashi, Koichi Koizumi, Souichi Yanamoto, Tomohiro Yamada, Masahiro Umeda

**Affiliations:** 1 Department of Oral Health, Nagasaki University Graduate School of Biomedical Sciences, Nagasaki, JPN; 2 Department of Oral and Maxillofacial Surgery, Nagasaki University Graduate School of Biomedical Sciences, Nagasaki, JPN; 3 Department of Oral Oncology, Graduate School of Biomedical and Health Sciences, Hiroshima University, Hiroshima, JPN

**Keywords:** bacterial count, intubation, povidone iodine, toothbrushing, ventilator-associated pneumonia

## Abstract

One of the causes of ventilator-associated pneumonia (VAP) is the aspiration of fluid-containing pathogenic bacteria into the lower respiratory tract. The purpose of this study was to determine whether oral povidone-iodine (PV-I) application reduces bacterial counts in the fluid on the cuff of an endotracheal intubation tube. Patients intubated and managed with tracheostomy for oral cancer surgery were divided into two groups. The PV-I group received a single topical application of PV-I in the oral cavity. The brushing group underwent tooth brushing with suction and cleaning. Before the intervention, and at one, two, three, and six hours after intervention, samples were taken from the mouth and the cuff to determine viable bacterial counts using the delayed real-time polymerase chain reaction method, which quantifies only viable bacteria. Seven patients in the PV-I group and six in the brushing group were included in the study. The brushing group showed an increase in bacterial counts in both the oral cavity and on the cuff up to six hours post intervention. In contrast, the PV-I group showed significantly lower bacterial counts from one to six hours post intervention, both in the oral cavity and on the cuff. These results indicate that topical PV-I application reduces the number of bacteria entering the lower respiratory tract and may help prevent VAP.

## Introduction

Although many bacteria and fungi exist in the oral cavity as commensal microorganisms, their numbers are kept in check by the self-cleaning function of the oral cavity and swallowing. However, in postoperative patients with oral cancer who are intubated and managed with tracheal intubation, the bacterial counts in the oropharyngeal fluid have been reported to increase >100-fold because the self-cleaning function of the oral cavity is significantly reduced [1,2]. The oral cavity is a breeding ground for pathogenic bacteria that can cause infections throughout the body [3,4]. The development of ventilator-associated pneumonia (VAP) is a problem in intubated patients, with one possible cause being the increased flow of oral bacteria into the lower respiratory tract [5]. Therefore, many attempts have been made to reduce the risk of developing VAP through oral care [6-8].

The Institute for Healthcare Improvement (IHI) advocates a ventilator bundle to prevent complications in patients on ventilators [9] and recommends daily oral care with 0.12% chlorhexidine (CHX) to prevent VAP. However, this concentration of CHX is contraindicated for oral use in Japan due to reports of cases of anaphylactic shock caused by CHX [10], and no oral care methods for intubated patients have been established. Therefore, we aimed to investigate whether oral care with povidone-iodine (PV-I), widely used as a mouthwash in Japan, is effective in preventing VAP as an alternative to 0.12% CHX. Povidone-iodine is a disinfectant that can be applied to mucous membranes and wounds, and it was chosen for this study because it is highly effective against a wide range of bacteria and has almost no side effects. The purpose of this preliminary study was to investigate the effect of oral application of PV-I on the number of bacteria entering the lower respiratory tract.

## Materials and methods

Participants

The subjects were patients with oral cancer who underwent tracheotomy, neck dissection, tumor resection, and reconstructive surgery with a free flap and managed under intubation at the Department of Oral and Maxillofacial Surgery, Nagasaki University Hospital or Hiroshima University Hospital, Japan, between December 1, 2023, and August 31, 2024. Patients with edentulous jaws, pregnancy or lactation, allergy to iodine, or abnormal thyroid function were excluded. As this was a preliminary study, the target number of cases was set at 20, without calculating the statistical sample size.

Allocation and intervention

This study is an open-label randomized controlled trial. Participants were randomized into two groups, the PV-I group and the brushing group, in a 1:1 ratio, using computer software. Oral care and sample collection were performed one or two days post surgery. For the PV-I group, the oral cavity was aspirated and wiped with a sponge brush, and then 5 ml of PV-I (ISODINE® SOLUTION 10%, Mundipharma Corporation, Tokyo, Japan) was soaked on a cotton ball and applied to the dorsum of the tongue and buccal mucosa. The brushing group received oral care with a toothbrush, using a sponge brush and suction.

Sample collection

Fluid in the oropharynx and above the tracheal cannula cuff was collected before and at one, two, three, and six hours after the oral care intervention with PV-I or brushing. Samples were collected using a 10 ml syringe, and cases where ≤0.5 ml was collected due to low fluid level on the cuff were excluded. Samples were frozen and stored until further analysis.

Delayed real-time polymerase chain reaction (DR-PCR)

The sample contained a mixture of viable and dead bacteria; hence, we used our novel DR-PCR method [11], which enables selective and quantitative analysis of only viable bacteria. The principle of this method is that a sample containing a mixture of viable and dead bacteria is first cultured in liquid, and only viable bacteria grow; then, real-time PCR is performed.

Samples from both groups were diluted three-fold in saline and sonicated to remove viscosity. First, a mixture of viable and dead bacteria was prepared. The viable bacteria were diluted 10 times with the collected sample. The dead bacteria were prepared by adding 7% povidone-iodine to the saliva sample to kill the bacteria. The dead bacteria were confirmed by culturing them anaerobically on BHI agar medium (Becton, Dickinson and Company, Franklin Lakes, New Jersey, United States) for 48 hours and confirming that no bacterial growth was observed. Samples with a ratio of viable bacteria to dead bacteria of 10:0, 5:5, 1:9, and 0:10 were prepared, diluted 10-fold, placed in BHI Infusion, and incubated in a liquid culture at 37°C for four hours using Anaeropak Kenki (Mitsubishi Gas Chemical Company, Inc., Tokyo, Japan). The number of bacteria in the samples after incubation was measured using real-time PCR, and a standard curve was created with the number of bacteria on the vertical axis and the percentage of viable bacteria on the horizontal axis. The samples to be measured were then diluted 10-fold, and incubated in liquid culture for four hours, and the number of bacteria was measured using real-time PCR to determine the percentage of viable and dead bacteria using the standard curve mentioned above as a reference. As the primers used in the real-time PCR, we used universal primers targeting 16rRNA (Forward: 5'-TCCTACGGGAGGCAGCAGT-3', Reverse:5'-GGACT ACCAGGGTATCTAATCCTGTT-3'), and the total bacterial count was measured. The details of the artificial DNA used in the real-time PCR, the reaction conditions, etc., followed the method we reported previously [12].

Statistical analysis

All statistical analyses were performed using IBM SPSS Statistics for Windows, Version 26.0 (Released 2019; IBM Corp., Armonk, New York, United States). Differences between pre- and post-intervention were examined using Mann-Whitney U-test. Statistical significance was set at p-value <0.05.

Ethics and registration

The study was performed in accordance with the 2013 Declaration of Helsinki and approved by the Clinical Research Review Board (CRB) of Nagasaki University (#CRB7180001). This study was conducted as a specific clinical study in accordance with Japan’s Clinical Trial Acts enacted in April 2018 in Japan. Written informed consent was obtained from all participants. The study protocol was registered in the Japan Registry of Clinical Trials (jRCT) on December 25, 2023 (jRCT1071230103).

## Results

Participants characteristics

Twenty patients were enrolled in the study. Five were excluded due to little or no aspiration and little or no fluid on the cuff, and one was excluded due to post-surgery illness and difficulty intervening. One patient was excluded after becoming edentulous due to surgery. As a result, seven patients (45-81 years old) were included in the PV-I group and six (37-81 years old) were included in the brushing group.

There were eight male and five female participants (mean age 65.4 years). The primary sites were the mandibular gingiva in six patients, the tongue in four, and the floor of the mouth, maxillary gingiva, and mandibular bone in one each. All patients were intubated and managed with tracheostomy (Table 1).

**Table 1:** Patient characteristics of the PV-I and brushing groups. PV-I: povidone-iodine; VC: vital capacity; FEV: forced expiratory volume

Factor		PV-I group (n=7)	Brushing group (n=6)	p value
Age, mean±SD	years	67.1±13.9	63.3±16.7	0.662
Sex, n	male	5	3	0.592
	female	2	3	
Primary site, n	tongue/floor of mouth	2	3	0.592
	others	5	3	
Hemoglobin, mean±SD	(g/dL)	13.0±1.50	13.4±2.26	0.722
Leukocytes, mean±SD	(/μL)	6242±1638	5125±1266	0.202
Lymphocytes, mean±SD	(/μL)	1624±6516	2178±1008	0.257
Albumin, mean±SD	(g/dL)	3.80±0.466	4.10±0.167	0.164
Creatinine, mean±SD	(mg/dL)	0.714±0.167	0.667±0.185	0.782
% VC, mean±SD		123±12.4	100±8.08	0.005
FEV 1.0%, mean±SD		78.7±5.33	77.1±2.37	0.522
Operation time, mean±SD	(minutes)	761±134	754±112	0.912
Blood loss, mean±SD	(g)	442±158	761±536	0.16

Bacterial count in the oropharyngeal fluid

Brushing Group

**Figure 1:**
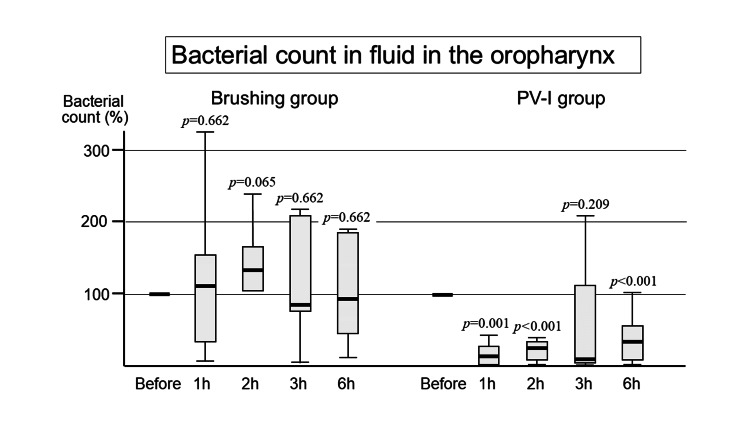
Bacterial count in the oropharyngeal fluid The number of bacteria tends to increase after tooth brushing although not significant, while it immediately decreases and the effect continues even after six hours in the PV-I group. PV-I: povidone iodine; h: hour

The percentage of viable bacteria post intervention is shown in Figure 1, taking the number of bacteria pre-intervention as 100%. One hour after brushing, the bacterial count increased, and then after two hours, it returned to a level almost the same as the value before the intervention, although there were no statistically significant differences.

PV-I Group

After the topical application of PV-I, the bacterial count in the oral cavity was immediately reduced. There were statistically significant differences at one, two, and six hours compared with pre-intervention (Figure 1).

Bacterial count in the fluid on the cuff

Brushing Group

**Figure 2:**
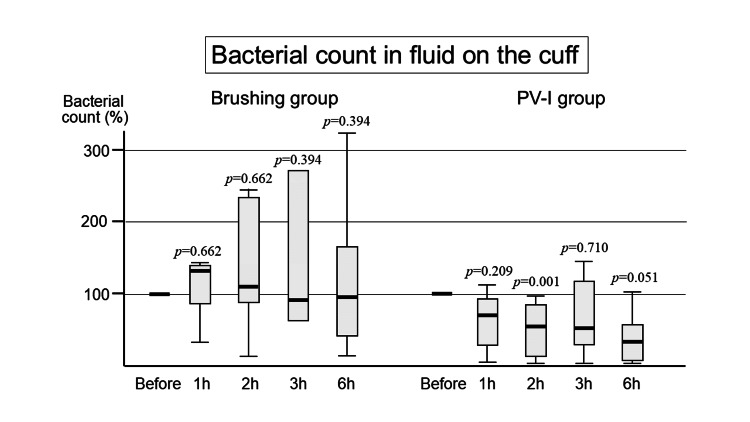
Bacterial count on the cuff Similar to the oropharyngeal fluid, the number of bacteria on the cuff also tends to increase in the brushing group. In contrast, it decreases until six hours later in the PV-I group. PV-I: povidone iodine; h: hour

Bacterial counts on the cuff increased at one hour, and then decreased to the value before intervention, although there were no statistically significant differences (Figure 2).

PV-I Group

After the topical application of PV-I, the bacterial count on the cuff decreased, similar to that in the oropharyngeal fluid. There were statistically significant differences between the bacterial counts before and at two hours after intervention (Figure 2).

## Discussion

VAP is a type of hospital-acquired pneumonia, that occurs in patients ventilated for >48 hours after the start of intubation. It has been reported to occur in 9-27% of intubated patients [13-15]. The mortality rate for VAP was 32.4%, which was higher than that for community-acquired pneumonia (6.3%), nursing-home healthcare-associated pneumonia (15.5%), and hospital-acquired pneumonia other than VAP (30.4%) [16]. It is thought to be caused mostly by pathogenic bacteria that enter the lower respiratory tract, and many attempts have been made to reduce the amount of bacteria that enter this tract.

In the ventilator bundle created by the IHI [6], the following were initially listed to prevent complications in intubated patients: (i) elevation of the head of the bed to between 30° and 45°, (ii) daily sedative interruption and daily assessment of readiness to extubate, (iii) peptic ulcer disease prophylaxis, (iv) deep venous thrombosis prophylaxis (unless contraindicated). Subsequently, a meta-analysis reported that applying 0.12% or 0.2% CHX to the oral cavity of intubated patients was associated with a decreased risk of developing VAP [17], and in 2010, (v) daily oral care with CHX was added. However, using this concentration of CHX in the oral cavity is contraindicated in Japan due to adverse events like anaphylaxis [10]. Therefore, no standard oral care technique has yet been established for intubated patients in Japan. It has been reported that brushing with a toothbrush decreases the number of oral bacteria if gargling is performed but conversely increases if gargling is not performed [18]. This is thought to be because the dental plaque adhering strongly to the tooth surfaces spreads into the mouth through tooth brushing, and saliva-containing bacteria cannot be completely collected without gargling. Since intubated patients cannot gargle, brushing may increase salivary bacterial counts, even when combined with suctioning and wiping. Several randomized clinical trials have reported that brushing does not prevent VAP [19-22]. The results of this study showed that brushing in combination with wiping and suctioning was not effective in reducing the number of bacteria in the oral cavity and on the cuff; on the contrary, the number of bacteria tended to increase. Although there was no significant difference from the viewpoint of preventing aspiration pneumonia, it is thought that toothbrushing is not necessary for intubated patients.

Based on these results, an alternative oral care technique to 0.12% CHX is needed for patients intubated in Japan. Therefore, we focused on PV-I, an antiseptic widely used in Japan. PV-I can be used in the oral cavity and has the strongest bactericidal action [23,24]. Few studies have evaluated the application of PV-I in the oral cavity to determine the risk of VAP compared to CHX. Chua et al. reported the first attempt to evaluate the efficacy of a PV-I oral wash in preventing VAP [25]. Intubated patients were randomly divided into two groups. The PV-I group received buccal swabs with 1% PV-I every eight hours from an intensive care unit (ICU) nurse. VAP occurred in six of the 22 patients in the PV-I group and in eight of the 20 patients in the control group. There were no statistical differences in VAP incidence, duration of intubation, or duration of ICU stay between the two groups. Seguin et al. also reported the efficacy of PV-I in preventing VAP [26]. They divided the intubated patients with head trauma into three groups: 36 patients receiving mouth rinse with 2.5% PV-I, 31 receiving mouth rinse with water, and 31 patients receiving control. The VAP incidence significantly decreased in the PV-I group, although the length of ICU stay and mortality did not significantly differ among the three groups. As described above, the effectiveness of the PV-I in preventing VAP is controversial. Labeau et al. concluded in a meta-analysis of 14 studies that VAP was significantly reduced in CHX studies, but the effect resulting from PV-I remains unclear because the result of PV-I was based on fewer studies and also showed a larger heterogeneity and broader confidence intervals [27].

Many medical practitioners in Japan have a negative view of the application of PV-I in the oral cavity. The first reason is the misconception that PV-I has tissue-damaging properties. In Japan, iodine tincture (an alcohol preparation of iodine with potassium iodide) has been widely used as a skin antiseptic. Iodine products such as iodine tincture or iodine glycerin, which are currently used to delineate precancerous lesions in the oral cavity and esophagus, are highly irritating when applied to the mucous membranes. However, PV-I has few side effects like tissue irritation, as evidenced by CDC guidelines recommending that wounds be cleaned with PV-I before closure to prevent surgical-site infection during surgical procedures. The second reason is the misconception that PV-I, when used in the oral cavity, causes xerostomia owing to its alcohol content. Although PV-I, like many other types of mouthwash, contains ethanol as an additive, the drug's information sheet states that it does not cause xerostomia, and a randomized clinical trial (RCT) reported that three months of rinsing with an alcohol-based mouthwash did not cause xerostomia [28]. To eliminate these misconceptions regarding PV-I, we are considering conducting a large-scale clinical study using the actual onset of VAP as an endpoint.

Although studies using the PV-I have set the incidence of VAP and mortality as endpoints, these are affected by a variety of patient conditions and studies with a larger number of patients are needed to confirm the effectiveness of oral care. Therefore, in this preliminary study, we examined the effect of oral care, with the number of bacteria on the cuff as the endpoint. A mixture of viable and dead bacteria is present in oropharyngeal fluid to which rinsing agents have been added. In real-time PCR, it is difficult to accurately evaluate changes in bacterial counts because the DNA of dead bacteria is also amplified. This effect was determined using DR-PCR, which is a combination of liquid culture and real-time PCR. The results showed that the number of bacterial cells entering the lower respiratory tract was significantly reduced in the PV-I group. This was thought to be due to the fact that PV-I applied in the oral cavity flowed into the lower respiratory tract and accumulated on the cuff, at the same time acting on the bacteria in the aspirated saliva. Future studies are needed to determine whether this directly contributes to a decrease in VAP; however, the present study showed that oral care using PV-I can reduce the number of bacteria entering the lower respiratory tract, which is one of the causes of VAP.

This study had several limitations. First, the number of cases was small for a preliminary study, and only postoperative patients were included; therefore, it is unclear whether the results are generalizable. Second, the endpoint was the change in the number of all bacteria cultured in the liquid medium, not the number of pathogenic bacteria; therefore, the risk of VAP and mortality could not be accurately evaluated. The primers used in this study can measure the total number of bacteria, but they do not react with fungi such as *Candida albicans*, so we have not been able to examine changes in fungi. However, since PV-I has a bactericidal effect on most microorganisms, including fungi, it is thought that it also reduces fungi that can cause pneumonia. In the future, it will be necessary to examine each type of bacteria, including fungi. Third, PV-I was administered only once, not continuously, in the oral cavity. Therefore, we could not confirm the effects or side effects of the long-term continuous PV-I application. For patients who cannot use PV-I due to allergy, we would like to consider using a different antiseptic mouthwash in the future. Furthermore, for patients who require long-term intubation, brushing is also necessary to prevent bacteremia caused by the worsening of periodontal disease, so we would also like to consider establishing an oral care method that combines brushing and PV-I. Future studies on VAP prevention should be conducted on a larger scale to determine its effectiveness based on VAP incidence and mortality rates.

## Conclusions

When toothbrushing using a sponge brush and suction was performed on intubated patients, the number of bacteria in the oropharyngeal fluid and on the cuff tended to increase. In contrast, following the topical application of PV-I, bacterial counts in the oral cavity and on the cuff decreased for over six hours post intervention. These findings suggest that oral care using PV-I may be effective in preventing VAP in intubated patients.
